# Total Oxidation of Toluene over Pt/CeO_2_-Fe_2_O_3_ Catalysts: Role of CeO_2_/Fe_2_O_3_ Ratio

**DOI:** 10.3390/nano16090507

**Published:** 2026-04-23

**Authors:** Anna Savel’eva, Diana Ponizovnaya, Grigory Mamontov

**Affiliations:** Research Laboratory of Porous Materials and Sorption, Tomsk State University, 36 Lenin Ave., Tomsk 634050, Russia; blokhina_as@mail.ru (A.S.); dianakirmas@gmail.com (D.P.)

**Keywords:** CeO_2_-Fe_2_O_3_, Pt, interphase, metal–support interaction, oxygen mobility, defects, toluene oxidation

## Abstract

This work examines the influence of the Ce/Fe ratio in Pt/CeO_2_-Fe_2_O_3_ catalysts on the peculiarities of metal–support interaction and catalytic properties in the total oxidation of toluene. The physical-chemical properties of the Pt/CeO_2_-Fe_2_O_3_ catalysts are studied using low-temperature N_2_ adsorption–desorption, XRD, TPR-H_2_, Raman, and TEM. The citrate method to synthesize the mixed CeO_2_-Fe_2_O_3_ supports makes it possible to obtain dispersed defective oxide particles that actively interact with the supported Pt species. An increase in the oxygen mobility of CeO_2_-Fe_2_O_3_ after the Pt deposition and the cooperation of active oxidative species with the active site of Pt is a key to the catalytic activity in the total oxidation of toluene. This effect is the highest for the Pt/3Ce2Fe catalyst, and the temperature of 50% toluene conversion over this catalyst is 167 °C.

## 1. Introduction

Increasing industrial and household emissions of volatile organic compounds (VOCs) pose a significant threat to the environment and human health [1]. Among the wide range of VOCs, aromatic hydrocarbons, particularly toluene, occupy a special place, being widely used as a solvent and fuel component. The toluene emissions into the atmosphere lead to the formation of photochemical smog and negatively impact human health, causing damage to the nervous and respiratory systems, causing a need to develop effective methods for its neutralization. One of the most effective methods for toluene neutralization is total catalytic oxidation using various types of catalysts [2,3,4].

Oxide systems such as perovskites (ABO_3_) [5], spinels (AB_2_O_4_) [6], and transition metal oxides (MnO_x_, Co_3_O_4_, CeO_2_) [7,8,9] can be used as catalysts for the VOC neutralization. However, a key role in the design of highly efficient oxidation catalysts is given to noble platinum group metals supported on oxide supports [10]. Platinum exhibits high activity in the cleavage of C–C and C–H bonds [11]; its catalytic properties and stability under harsh reaction conditions critically depend on the nature of the support and the phenomenon occurring at the phase interface, known as strong metal–support interaction [12,13,14].

The catalytic activity of systems is determined by multiple factors, one of which is the mobility of lattice oxygen in the oxide support. CeO_2_ is an important support for heterogeneous catalysis due to its redox ability associated with a facile transition between the Ce^4+^ and Ce^3+^ states, which is accompanied by the formation of oxygen vacancies and high mobility of lattice oxygen. The density of oxygen vacancies in CeO_2_ critically affects catalytic processes involving oxygen, such as the oxidation of CO and VOCs [15]. As shown in a number of studies, the formation of the Pt–O–Ce interface structures on the CeO_2_ surface not only stabilizes the Pt clusters but also creates active oxygen activation sites that intensify the toluene oxidation via the Mars-van Krevelen mechanism [16]. Moreover, recent studies demonstrate the possibility of fine-tuning the SMSI strength in the Pt/CeO_2_ systems by modifying the surface or creating a defective structure, which directly affects the electronic state of Pt and its catalytic activity. Iron oxide (Fe_2_O_3_) has attracted attention as a more economically affordable and thermally stable support, also possessing pronounced redox activity. Methods to create defects in the Fe_2_O_3_ supports [17] were described in the literature, enabling strong electronic interactions with the supported platinum, facilitating the formation of charged Pt sites and the activation of both molecular oxygen [18] and oxygen in the iron oxide lattice. In Ref. [18], the role of the Pt–Fe sites in O_2_ activation in the oxygen reduction reaction was demonstrated. A single-site Pt–Fe pair was synthesized using a photochemical deposition method on α-Fe_2_O_3_, and H_2_PtCl_6_ and methanol were used as a platinum source and a sacrificial agent, respectively. It was shown that due to the electron interaction between platinum and iron, this catalyst had partially filled orbitals, and the Pt–Fe pair cooperatively adsorbed O_2_ according to the lateral interaction model and dissociated the O=O bond.

The introduction of a second metal/transition metal oxide (Zr, Mn, Fe, etc.) to ceria leads to an increase in the defectiveness of its structure and the number of oxygen vacancies, which is critically important in catalytic oxidation reactions [19,20,21]. For iron oxides, an increase in the mobility of the lattice oxygen can be achieved by creating α-Fe_2_O_3_/γ-Fe_2_O_3_ interphase boundaries, at which asymmetrically coordinated mobile oxygen of the crystal lattice participating in oxidation processes is formed [22].

Thus, the synthesis of the CeO_2_-Fe_2_O_3_ mixed oxide systems allows not only to combine the advantages of both oxides but also to create new interfacial sites that synergistically enhance oxygen transport [23] and stabilize active Pt species. The Fe^3+^ introduction into the CeO_2_ lattice or the formation of an interface between the oxides can distort the crystal lattice, increase the concentration of oxygen vacancies, and facilitate the migration of active oxygen species to toluene adsorption sites [24]. Despite the potential of the CeO_2_-Fe_2_O_3_ mixed oxides, systematic studies of the influence of the oxide ratio, method of preparation and the nature of their interaction with platinum on the catalytic properties in the total oxidation of toluene are insufficiently presented in the literature. In addition, H_2_PtCl_6_ is widely used as a precursor of platinum, while in the present study, the (Me_4_N)_2_[Pt_2_(OH)_2_(NO_3_)_8_] was used as a Pt precursor [25]. Pt/CeO_2_ catalysts were prepared using this Pt complex in [25], while Pt/FeO_x_ was not synthesized.

The aim of the present work is to synthesize by the citrate method individual CeO_2_ and Fe_2_O_3_ and mixed Ce-Fe oxides with different Ce/Fe ratios, as well as Pt catalysts based on these, and to establish the features of this mixed oxide structure and the features of the Pt interaction with the surface of oxides and catalytic properties in total oxidation of toluene. The formation of mixed Ce-Fe oxides, as well as CeO_2_-Fe_2_O_3_ interfaces, and the cooperation of redox sites on the support surface with Pt active species are discussed. The complex of physical-chemical methods is used to study the features of porous structure (N_2_ adsorption–desorption), the structure (XRD, TEM, Raman) and features of the Pt-support interaction, and the cooperation of the active site of platinum with the oxidative site of CeO_2_ and FeO_x_ phases of support (TPR-H_2_). The structure–activity relationship was established.

## 2. Materials and Methods

### 2.1. Synthesis of Supports and Catalysts

Oxide supports designated as CeO_2_, 3Ce2Fe, 1Ce6Fe, and Fe_2_O_3_ were prepared by the citrate method using Ce(NO_3_)_3_*6H_2_O and Fe(NO_3_)_3_*9H_2_O (chemically pure grade, Reakhim) as metal oxide precursors and citric acid (CA) as a complexing agent (reagent grade, Reakhim). A constant molar ratio of the total amount of metal cations to citric acid was specified for all syntheses: Ʃn(Me):n(CA) = 1:2. To synthesize the individual oxides, CeO_2_ and Fe_2_O_3_, the 0.4 M CA solution was added to a 0.2 M solution of the corresponding precursor. To synthesize the mixed oxide systems, a weighed sample of Ce(NO_3_)_3_*6H_2_O was dissolved to form 50 mL of a solution, then 50 mL of Fe(NO_3_)_3_ solution was added with stirring. Then, the CA solution was added to the solution of the metal nitrates, and the pH of the solution was 1. The mixed oxide systems had the following molar compositions: 3Ce2Fe—n(Ce):n(Fe) = 3:2 (corresponding to 75% CeO_2_—25% Fe_2_O_3_) and 1Ce6Fe—n(Ce):n(Fe) = 1:6 (corresponding to 25% CeO_2_—75% Fe_2_O_3_). The solutions were stirred at room temperature for an hour at 500 rpm, then the temperature was gradually raised to 90 °C. At 90 °C, the solution was aged for 1.5–2 h until a gel formed. The samples were then dried at 120 °C for 10 h and calcined at 450 °C for 4 h.

To support Pt onto the surface of the obtained supports, a solution of (Me_4_N)_2_[Pt_2_(OH)_2_(NO_3_)_8_] [25] in acetone was used based on 2 wt.% Pt in the catalyst. The resulting Pt-containing catalysts were dried at 100 °C for 8 h and calcined in an air atmosphere at 400 °C for 4 h. The resulting catalysts Pt/CeO_2_, Pt/3Ce2Fe, Pt/1Ce6Fe, and Pt/Fe_2_O_3_ were studied using a range of physical-chemical analytical methods as well as in the total oxidation of toluene.

### 2.2. Study of the Physical-Chemical Properties of Catalysts

Textural characteristics of the samples were analyzed using low-temperature N_2_ adsorption on the 3Flex analyzer (Micromeritics, Norcross, GA, USA). The specific surface area (S_BET_) of the catalysts was determined using the BET method. Pore size distribution was calculated using the BJH method based on the desorption branch of the isotherm.

The surface reactivity of the samples was studied using the temperature-programmed reduction in hydrogen (TPR-H_2_) on the AutoChem 2950 HP analyzer (Micromeritics, Norcross, GA, USA). The samples were pre-oxidized in an air flow of 20 mL/min at 400 °C for 10 min at a heating rate of 10 °C/min, then cooled to 25 °C and reduced in the 10% H_2_/Ar mixture at a flow rate of 20 mL/min to a temperature of 900 °C with a heating rate of 10 °C/min.

The phase composition of the supports and catalysts was analyzed using the Shimadzu 7000 diffractometer with the monochromatic CuK_α_ radiation in the 2θ range of 20–80°. The phase composition of the samples was identified using the PDF-2 database. The CSR of the metal oxides was calculated using the Williamson–Hall method in the Powder-Cell 2.5 software package.

The morphology, crystal structure, and local chemical composition of the samples were studied using the high-resolution transmission electron microscopy (HRTEM) on the THEMIS Z microscope (Thermo Fisher Scientific, Waltham, MA, USA) operating at an accelerating voltage of 200 kV and a limiting resolution of 0.07 nm. Images were taken using the Ceta 16M™ digital camera. The elemental composition was analyzed using the SuperX energy-dispersive spectrometer (EDX, Thermo Fisher Scientific, Waltham, MA, USA) equipped with a silicon semiconductor detector with an energy resolution of 128 eV. For microscopic studies, sample powders were dispersed in the alcohol suspensions and applied to carbon substrates with perforated grids using an ultrasonic disperser.

Raman spectroscopy was used to study the defect structure of as-prepared samples. Spectra were recorded using the InVia confocal Raman spectrometer (Renishaw, Wharton, UK) equipped with a 785 nm Nd:YAG laser operating at 100 mW. The spectral range and resolution were 190–1000 cm^−1^ and 2 cm^−1^, respectively.

### 2.3. Study of Catalytic Properties of Samples in Total Oxidation of Toluene

The catalytic properties of the CeO_2_-Fe_2_O_3_ oxide systems and Pt catalysts based on these were studied in the total oxidation of toluene using the flow-through catalytic system (Catacon, Novosibirsk, Russia) with online detection of the oxidation products using a thermal catalytic sensor. Toluene oxidation was carried out in a flowing model gas mixture containing 20% O_2_, 79.9% N_2_, and 0.1% (or 1000 ppm) toluene. The catalyst volume of the 0.25–0.5 mm fraction was 0.2 cm^3^, and the flow rate was 100 mL/min (30,000 h^−1^). The reactor and catalyst were preheated in a mixture of 20% O_2_ and 80% N_2_ to 350–400 °C, after which toluene was added. Toluene concentrations at the reactor inlet were determined using the Kristall 5000.2 (Chromatec instruments, Yoshkar-Ola, Russia) gas chromatograph equipped with a flame ionization detector (FID). To prevent the toluene chemisorption at low temperatures [26], the catalytic reaction was conducted with cooling from 400 °C to 25 °C at a rate of 10 °C/min. After the CO_2_ signal reached a steady state, and the concentration of toluene was zero according to gas chromatography, the furnace temperature was decreased at a rate of 10 °C/min. The resulting temperature dependences of CO_2_ yield were converted into the values for the toluene conversion to CO_2_. During the catalytic reaction, gas chromatography revealed that at low temperatures, a small amount of byproducts, such as benzaldehyde, appeared. The maximum total amount did not exceed 2–5%, which was within the chromatographic error limits. The carbon dioxide concentration at the reactor outlet was measured every 5 s using a thermal catalytic sensor (Test1, Gasanalizator, Smolensk, Russia) with internal calibration. The toluene conversion to CO_2_ was then calculated as:

$$X(toluene\;to\;{CO}_{2})=\;\frac{n({CO}_{2})}{7\times n(toluene)}\times 100\%$$

## 3. Results

**Figure 1:**
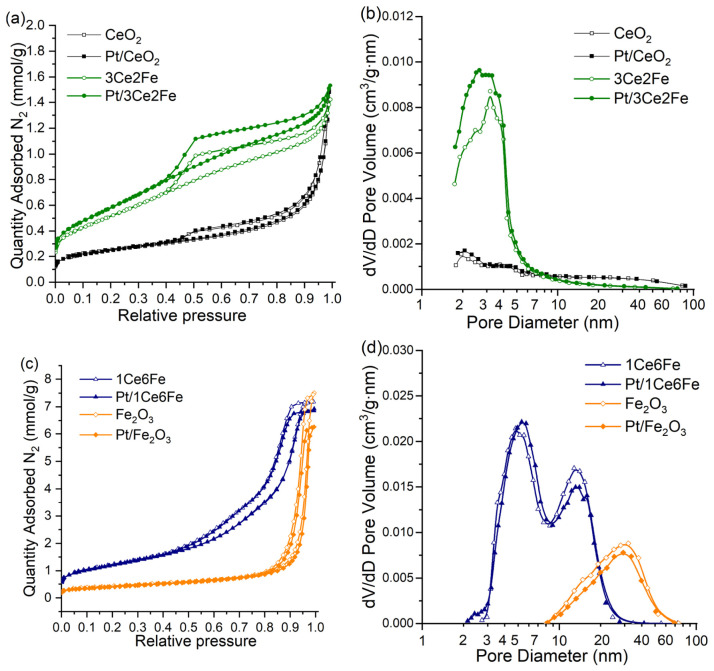
Nitrogen adsorption–desorption isotherms (**a**), (**c**) and pore size distributions (**b**), (**d**) for oxide supports and the Pt catalysts based on them.

Figure 1a shows the low-temperature nitrogen adsorption–desorption isotherms for the oxide supports and Pt catalysts based on these. All samples are mesoporous materials; however, the pore size distribution pattern varies depending on the chemical composition of the support. Ceria is characterized by a relatively low specific surface area (19 m^2^/g), the pore size distribution lies in the region of narrow mesopores (up to 5 nm), and a small number of wide pores of 10–80 nm in size are also present. The 3Ce2Fe sample has a wider hysteresis loop in the low-temperature nitrogen adsorption–desorption isotherm, and the pore size distribution also lies in the region of up to 5 nm. The specific surface area of the 3Ce2Fe sample increases (48 m^2^/g) compared to CeO_2_ (Table 1).

The textural properties of the Fe_2_O_3_ and 1Ce6Fe oxide systems (Figure 1c) differ significantly from those of CeO_2_ and 3Ce2Fe. The nitrogen adsorption–desorption isotherms of Fe_2_O_3_ also belong to type IV according to the IUPAC classification but are characterized by significantly higher nitrogen adsorption as well as by a pronounced hysteresis loop in the relative pressure range from 0.8 to 1. The sample has a broad pore size distribution with a maximum at ~27 nm. The 1Ce6Fe sample is characterized by a bimodal pore size distribution with maxima at 5 and 15 nm, which may be due to the formation of the ceria sites with narrow mesopores and iron oxide sites with wider mesopores. Moreover, the 1Ce6Fe sample has the largest specific surface area, i.e., 96 m^2^/g.

After Pt deposition, the specific surface area and pore volume for Pt/CeO_2_ remained unchanged. For Pt catalysts supported on 3Ce2Fe and 1Ce6Fe mixed oxide supports and on Fe_2_O_3_, a slight change in specific surface area was observed.

**Figure 2:**
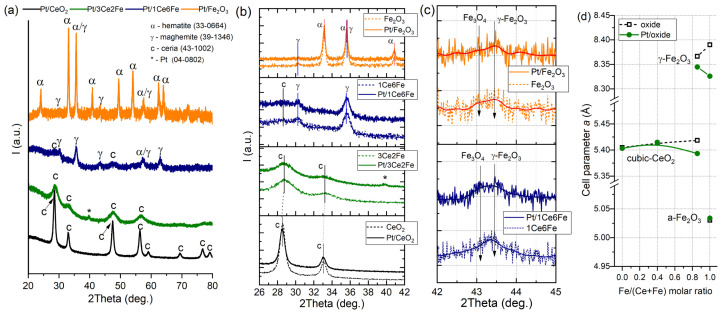
Diffraction patterns of supported Pt catalysts (**a**), corresponding fragments of patterns for supports and catalysts at 26–42° 2Theta (**b**) and 42–45° 2Theta (**c**). Dependences of cell parameter *a* for supports and catalysts (**d**).

Figure 2 shows the phase composition of the Pt catalysts deposited on the Ce-Fe oxide supports. The diffraction pattern of Pt/CeO_2_ contains reflections corresponding to the cubic phase of ceria with a fluorite structure (PDF card 43-1002). The CeO_2_ structure is formed by well-crystallized crystallites with the CSR size of ~17 nm (Table 1). With the introduction of 25% Fe_2_O_3_ into the support composition (Pt/3Ce2Fe sample), a decrease in the intensity and a broadening of reflections corresponding to CeO_2_ are observed, indicating an increase in the dispersion of the ceria particles (CSR size of ~9 nm); no clear reflections of the Fe-containing phases were detected, indicating high dispersion of the iron oxide component. The low-intensity peaks of γ-Fe_2_O_3_ (PDF card 39-1346) and Fe_3_O_4_ (PDF card 19-0629) were found (Figure 2b,c). The Fe introduction into the oxide support leads to an increase in the lattice parameter of CeO_2_ (Table 1 and Figure 2d), while the magnitude of lattice micro-distortions (Δd/d) increases sharply, which may be due to the partial incorporation of iron into the CeO_2_ crystal lattice. Most likely, due to the significant difference in the ionic radii of cerium and iron (r(Ce^4+^) = 0.097 nm, r(Fe^3+^) = 0.064 nm), a solid interstitial solution of iron in CeO_2_ is formed. However, in Ref. [27] it was shown that the microstructure of fluorite Ce_x_Fe_1−x_O_2_ solid solutions, obtained by the coprecipitation of Ce and Fe nitrate solutions, was determined by the amount of doping Fe^3+^ ions. At a low molar ratio of Fe (<0.15), Fe^3+^ occupies both the Ce^4+^ lattice positions and the interstitial sites; however, doping occurs exclusively in the bulk only at Fe concentrations below 0.04. It extends to the surface region with a high content of Fe ions (from 0.04 to 0.13). At a high Fe molar ratio (from 0.2 to 0.3), the Fe-O coordination begins to transform into the sub-Fe_2_O_3_ units in the solid solution, and eventually, bulk α-Fe_2_O_3_ is formed when the Fe molar ratio reaches 0.3. In addition, the calcination temperature plays an important role in the formation of the solid solution, affecting the Fe-O coordination structure in the fluorite lattice. In the case of the 3Ce2Fe sample obtained at a lower calcination temperature (450 °C), the iron oxide remains in an X-ray amorphous state; segregation of the bulk Fe_2_O_3_ phase as well as the formation of a massive solid solution of iron in CeO_2_ does not occur.

The Fe_2_O_3_ sample is a mixture of α- and γ-Fe_2_O_3_ phases with the CSR sizes of 36 and 17 nm, respectively. With the addition of 25% CeO_2_ (1Ce6Fe and Pt/1Ce6Fe samples), mutual dispersion of Ce and Fe oxides occurs. The crystallite size of CeO_2_ is 9 nm, and that of γ-Fe_2_O_3_ is 16 nm. In the presence of CeO_2_, there is no α-Fe_2_O_3_ phase, and the mixture of γ-Fe_2_O_3_ and Fe_3_O_4_ phases is formed (Figure 2c). In Ref. [28], it is shown that a decrease in the crystallite size lowers the phase transition temperature of iron oxide. In the case of the 1Ce6Fe sample, despite the decrease in the crystallite sizes of the oxide phases, the phase transition from γ-Fe_2_O_3_ to α-Fe_2_O_3_ does not occur. It is known that the introduction of Pt and CeO_2_ to Fe_2_O_3_ inhibits the growth of the iron oxide crystallinity at a temperature of 400 °C, facilitates the preservation of the mesoporous space of the samples, and also increases the thermal stability of the Pt catalysts [29]. In Ref. [30], the effect of the amount of citric acid (Ʃn(Me):n(LA) varied from 1:1 to 1:1.3) on the phase composition of CeO_2_-Fe_2_O_3_ composites with different Ce-Fe ratios was investigated. The authors found that lower concentrations of citric acid led to higher crystallinity in the oxides. In our work, a twofold excess of citric acid with respect to the number of moles of metals was used. Thus, several factors simultaneously determined the high dispersion and phase composition of 1Ce6Fe: a relatively low calcination temperature, a small amount of CeO_2_ in the composition, and an excess of citric acid taken at the synthesis stage.

After the Pt deposition, the phase composition and phase ratio according to the XRD data remain unchanged (Table 1). However, due to the high dispersion of the oxides, it is impossible to unambiguously assess the Pt effect on the phase composition of the mixed oxide samples. For the Pt/3Ce2Fe sample, a reflection of metallic Pt (111) appears at 39.8°. For other Pt catalyst samples studied, no Pt-related reflections are detected, which is due to its low content and highly dispersed state on the support surface. According to the analysis of the cell parameter changes after the Pt deposition (Figure 2d), one can conclude that CeO_2_ and α-Fe_2_O_3_ phases are not changed significantly, which may be connected with their high stability, large crystalline sizes, and/or weak interaction with Pt. However, a significant decreasing of the cell parameters of the γ-Fe_2_O_3_ phase and CeO_2_ phase (for Pt/1Ce6Fe) for the Pt catalysts in comparison with the oxide supports is observed. This indicates participation of this phase in the Pt stabilization, a possible redox reaction of the Pt precursor with the surface, with the corresponding changes in Fe^2+^/Fe^3+^ and Ce^3+^/Ce^4+^ ratio in γ-Fe_2_O_3_/Fe_3_O_4_ and CeO_2_ dispersed phases.

Thus, according to the XRD results, the increased dispersion of both CeO_2_ and FeO_x_ phases is observed for mixed CeO_2_-Fe_2_O_3_ support. Both Fe incorporation into the CeO_2_ phase and the formation of CeO_2_-Fe_2_O_3_ interfaces are observed. Ceria ensures the stabilization of Fe_3_O_4_ and γ-Fe_2_O_3_ phases in mixed CeO_2_-Fe_2_O_3_ oxides. The platinum is distributed in a highly dispersed state; the reflections of metallic Pt are observed only for the Pt/3Ce2Fe catalyst.

**Figure 3:**
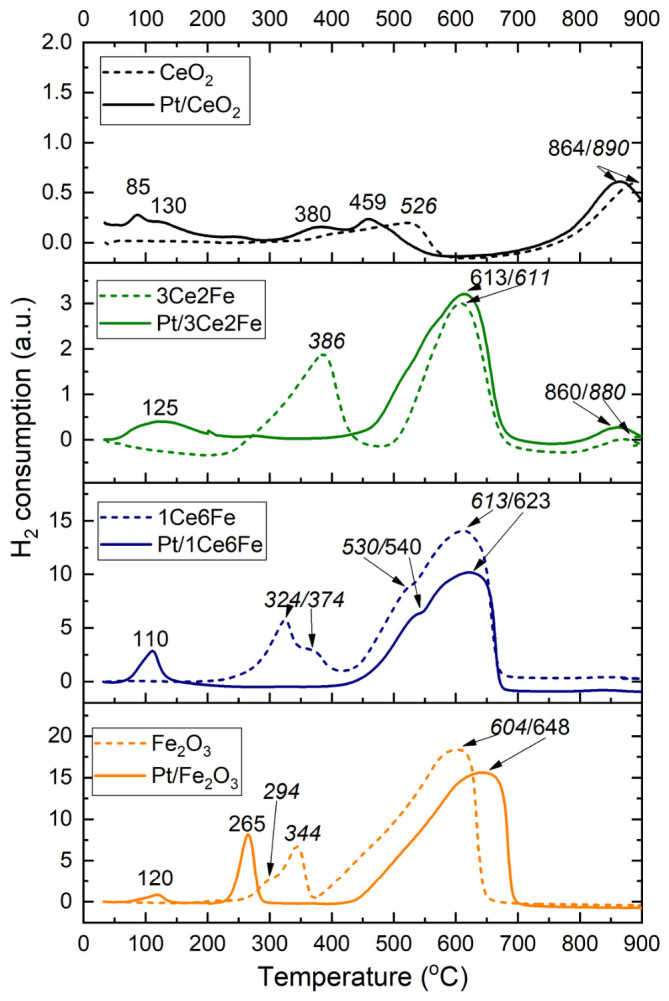
TPR-H_2_ profiles of oxide Ce-Fe supports and Pt catalysts based on them.

To evaluate the surface reactivity of the supports and catalysts, temperature-programmed reduction experiments with hydrogen were conducted. Figure 3 shows the TPR-H_2_ profiles for the supports and Pt catalysts. The TPR profile of CeO_2_ exhibits a maximum at 526 °C and a high-temperature hydrogen consumption peak (>750 °C), corresponding to the reduction in surface lattice oxygen and oxygen from the bulk of the CeO_2_ phase [31]. The profile of Fe_2_O_3_ is characterized by a stepwise reduction pattern of iron oxides [32], including the following stages: Fe_2_O_3_ → Fe_3_O_4_ (at temperatures from 200 °C to 400–450 °C), Fe_3_O_4_ → FeO → Fe (at temperatures above 400–450 °C). The broadening of the low-temperature hydrogen consumption peak with a maximum at 344 °C and the appearance of a shoulder at 294 °C are probably due to the difference in the processes of surface and bulk reduction in Fe_2_O_3_ particles. In Ref. [33], during the reduction in the surface of Ag/FeO_x_ catalysts under TPR-H_2_ conditions, the appearance of a broad peak with maxima at 320 °C and 350 °C was observed, which the authors attributed to the reduction in the surface and bulk of Fe_2_O_3_, respectively. The intensity ratio of these maxima indicated the predominance of the surface reduction of iron oxide particles, which was consistent with the highly dispersed state according to the XRD data. In the present work, the Fe_2_O_3_ sample is coarsely dispersed; the ratio of the reduction maxima at 294/344 °C also indicates the predominance of the share of bulk reduction over the particle surface.

The Fe introduction (3Ce2Fe sample) significantly changes the TPR profile of ceria. A broad hydrogen consumption maximum from 225 °C to 450 °C with a maximum at 386 °C corresponds to the first stage of the Fe_2_O_3_ reduction, as well as the reduction in surface oxygen species in the distorted CeO_2_ lattice. In Ref. [34], doping CeO_2_ with iron led to a significant shift in the peak of the surface oxygen reduction in CeO_2_ towards lower temperatures. This result was consistent with a significant decrease in the O binding energy upon replacing Ce^4+^ on the surface with Fe^3+^, as was shown by the DFT method. The TPR profile also contains an intense hydrogen consumption maximum at 611 °C, corresponding to the second stage of the iron oxide reduction, i.e., Fe_3_O_4_→FeO (Fe). The Fe introduction does not affect the position of the high-temperature peak of oxygen reduction (880 °C) from the bulk of the CeO_2_ lattice.

A complex reduction profile is observed for the 1Ce6Fe sample. In the range from 200 to 420 °C, as in the case with the 3Ce2Fe sample, the reduction in Fe_2_O_3_ and the CeO_2_ surface occurs. For the 1Ce6Fe sample, the “low-temperature” reduction peak has maxima at 324 °C and 374 °C, which may correspond to the reduction in the surface and bulk of finely dispersed Fe_2_O_3_ particles to Fe_3_O_4_. The high-temperature hydrogen consumption maximum at 613 °C remains virtually unchanged compared to the 3Ce2Fe sample, but a more pronounced shoulder appears at 530 °C. Thus, the Fe introduction into the oxide support facilitates the activation of the CeO_2_ surface reduction process due to the formation of interstitial surface solid solutions and an increase in the ceria defectiveness. Moreover, the ceria introduction into the composition of iron oxide leads to an increase in its dispersion but does not have a qualitative effect on the behavior of the reduction of iron oxides.

The TPR-H_2_ profiles of the supported Pt catalysts are different from those of the respective oxide supports. The reduction profile of the Pt/CeO_2_ sample exhibits a maximum at 85 °C, corresponding to the reduction in dispersed PtO_x_ sites weakly bound to the CeO_2_ surface [35]. With the increase in the reduction temperature, the strength of the bond between PtO_x_ and the surface increases; the shoulder of the consumption peak at 130 °C can probably be attributed to the reduction in smaller PtO_x_ clusters, or, as shown in Ref. [36], the reduction peak for 1 Pt/CeO_2_-450 was attributed to the hydrogenation of oxygen atoms near the single-atom Pt^2+^ sites. At temperatures above 300 °C, maxima appear at 379 and 462 °C, which can be attributed to the Pt reduction from single-atom states or the Ce_1−x_Pt_x_O_2−y_ solid solution [37], as well as to the reduction in the CeO_2_ surface.

The reduction in Pt/Fe_2_O_3_ is characterized by a broad hydrogen consumption peak with a shoulder at ~90 °C and a maximum at 120 °C, corresponding to the reduction in the dispersed PtO_x_ sites. A narrow hydrogen consumption maximum at 265 °C also appears on the profile. In Ref. [38], a similar reduction pattern was observed for the Pt/α-Fe_2_O_3_ with a maximum at 128 °C, related to the PtO_x_ reduction to Pt^0^, and at 277 °C, related to the reduction in α-Fe_2_O_3_ to Fe_3_O_4_ in the presence of platinum.

The reduction patterns of Pt/3Ce2Fe and Pt/1Ce6Fe are similar. The high-temperature reduction region of the catalysts (above 400 °C) is virtually identical to the reduction profiles of the supports described above. The low-temperature region exhibits broad hydrogen consumption maxima at 123 °C and 110 °C, respectively, for Pt/3Ce2Fe and Pt/1Ce6Fe, characterizing the reduction in various dispersed PtO_x_ sites, as well as the reduction in surface oxygen species in CeO_2_. Unlike the TPR profiles for the oxide supports, the Pt/3Ce2Fe and Pt/1Ce6Fe catalyst profiles lack the low-temperature peak of Fe_2_O_3_→Fe_3_O_4_ reduction in the range from 200 °C to 400 °C. It is likely that the dispersed Fe_2_O_3_ phase is reduced to Fe_3_O_4_ already during the Pt deposition. The presence of the Fe_3_O_4_ phase for Pt/3Ce2Fe catalysts was shown by XRD (Figure 2c). A similar reduction pattern was observed for Fe_3_O_4_ and Pt/Fe_3_O_4_ catalysts in Ref. [39]. In the presence of platinum, the reduction temperature of the Pt/Fe_2_O_3_ surface decreased, which the authors explained through the strong Pt interaction with the iron oxide surface and, as a consequence, the weakening of the Fe–O bond energy. In the Pt/3Ce2Fe and Pt/1Ce6Fe samples, it is likely that the strong Pt interaction with the surface of highly dispersed CeO_2_ and iron oxide is not the only one that occurs.

The Pt/Fe_2_O_3_ reduction pattern differs from those for Pt/3Ce2Fe and Pt/1Ce6Fe reduction profiles. The Pt presence similarly lowers the iron oxide reduction temperature, but the increase in Fe_2_O_3_ crystallite size and the absence of the second component, CeO_2_, do not lead to the complete reduction in Fe_2_O_3_ to Fe_3_O_4_ during the Pt deposition.

Thus, it can be concluded from TPR-H_2_ results that Pt significantly increases the reducibility of both FeO_x_ and CeO_2_ phases. This can lead to the cooperation of oxidative species on the Pt and support surfaces. The high growth of the low-temperature (50–250 °C) reaction ability was demonstrated for Pt/CeO_2_ and Pt/3Ce2Fe catalysts, while for the Fe-rich catalysts, the low-temperature reaction ability of the support decreases because of Fe_3_O_4_ phase formation as well as possible strong bonding of single-atom Pt species on the iron oxide.

**Figure 4:**
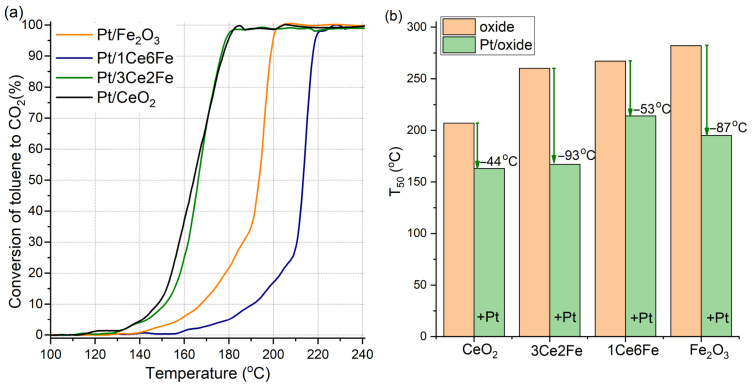
Toluene to CO_2_ conversion curves on Pt catalysts (**a**), comparison of T_50_ for oxide supports and Pt catalysts based on them (**b**).

The catalytic properties of the oxide supports and Pt catalysts were studied in the total oxidation of toluene. The selectivity toward CO_2_ during toluene conversion over Pt catalysts was close to 100%, while a small amount of by-products such as benzaldehyde was detected by gas chromatograph at temperatures of 130–180 °C. Figure 4a shows the curves of the toluene conversion into CO_2_ over Pt catalysts. For Pt/CeO_2_ and Pt/3Ce2Fe samples, the toluene conversion begins at ~130 °C, and the 50% conversion temperatures also differ slightly, i.e., 163 °C and 167 °C, respectively (Figure 4b). Upon reaching 60% conversion, the curves for the two samples become virtually indistinguishable. Complete toluene conversion over Pt/CeO_2_ and Pt/3Ce2Fe is achieved at a temperature of ~180 °C.

Toluene conversion on Pt/Fe_2_O_3_ and Pt/1Ce6Fe catalysts begins at higher temperatures. For the Pt/Fe_2_O_3_ and Pt/1Ce6Fe catalysts, the onset temperatures of toluene conversion are 142 °C and ~160 °C, respectively.

Figure 4b shows a comparison diagram of the 50% conversion temperature of toluene oxidation for the oxide supports and Pt catalysts based on them. For pure CeO_2_ and Fe_2_O_3_, the T_50_ values are 207 °C and 282 °C. For mixed oxide supports, the T_50_ values are slightly lower than for Fe_2_O_3_, reaching 267 °C and 260 °C for 1Ce6Fe and 3Ce2Fe, respectively. The diagram shows that the T_50_ value increases with the increasing Fe_2_O_3_ content in the CeO_2_-Fe_2_O_3_ support. The Pt introduction on the oxide surface leads to significant decreasing of the T_50_ value. The ∆T_50_ is −44 °C for Pt/CeO_2_ in comparison with pure CeO_2_, while the highest effect is observed for Pt/3Ce2Fe catalysts (∆T_50_ = −93 °C in comparison with 3Ce2Fe sample).

In Ref. [24], the Pt/CeO_2_-Fe_2_O_3_ catalysts for toluene oxidation were described, in which Pt facilitated an increase in the mobility of lattice oxygen in the support. For the Pt/CeO_2_ and Pt/3Ce2Fe catalysts, toluene oxidation likely also proceeds via the Mars-van Krevelen mechanism. For the Pt/1Ce6Fe catalyst, the oxygen transfer process is hindered due to the highly disordered structure of the support. The X-ray amorphous ceria in the presence of iron oxide does not form a bulk crystalline site, and the concept of lattice oxygen cannot be applied in this case.

Thus, the increased catalytic activity of the Pt catalysts compared to the oxide supports can be explained by both the increased amount of mobile lattice oxygen and the creation of interphase boundaries between Fe_2_O_3_, CeO_2_, and Pt. To establish the mutual influence of oxides and Pt on catalyst defects, the samples of supports and Pt catalysts were studied using Raman spectroscopy.

**Figure 5:**
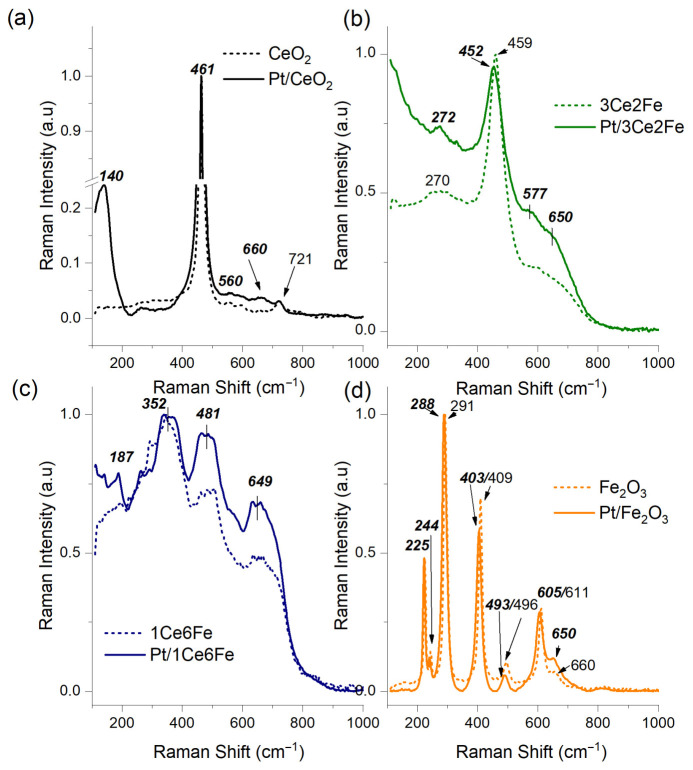
Raman spectra (λ_ex_ = 785 nm) of oxide supports and Pt catalysts based on them: (**a**) CeO_2_ and Pt/CeO_2_; (**b**) 3Ce2Fe and Pt/3Ce2Fe; (**c**) 1Ce6Fe and Pt/1Ce6Fe; and (**d**) Fe_2_O_3_ and Pt/Fe_2_O_3_.

Figure 5 shows the Raman spectra of the oxide supports and Pt catalysts. The spectrum of CeO_2_ contains a high-intensity band at 463 cm^−1^, which corresponds to the F_2g_ vibration mode of the cubic fluorite structure of CeO_2_ [40]. With the introduction of iron into the support, a slight shift in the F_2g_ mode toward lower wavenumbers is observed for the 3Ce2Fe sample, which may indicate the incorporation of Fe ions into the fluorite lattice [41]. In Ref. [42], a broadening of the F_2g_ band and a shift in the maximum for MnO_x_-CeO_2_ systems obtained by the solvothermal method were observed and explained by an increase in the dispersion and defectiveness of ceria in the presence of highly dispersed X-ray amorphous manganese oxide. The increased level of structural defectiveness of CeO_2_ in the presence of iron is also evident from the appearance of a broad band in the region of 500–700 cm^−1^, whereas this band is absent from the spectrum of pure CeO_2_.

The prominent peak at 225 cm^−1^ in the Fe_2_O_3_ spectrum corresponds to the A_1g_ vibrational mode in Fe_2_O_3_, which is associated with the symmetrical stretching vibrations of the Fe–O bonds in the hematite phase. Similarly, the peaks at 244 cm^−1^ (E_g_), 291 cm^−1^ (E_g_), 409 cm^−1^ (E_g_), 495 cm^−1^ (A^1g^), and 611 cm^−1^ (E_g_) are characteristic vibrational modes of Fe_2_O_3_, indicating its crystalline structure [43]. The shoulder at 660 cm^−1^ can likely be attributed to γ-Fe_2_O_3_ impurities, the presence of which was detected by the XRD. The γ-Fe_2_O_3_ phase is a cation-deficient spinel crystal structure with the formula (Fe^3+^)[Fe_5/3_^3+^,□_1/3_]O_4_, where () and [] denote the tetrahedral and octahedral positions, respectively, and □ denotes the iron cation vacancies. According to XRD data, the 1Ce6Fe sample is a mixture of dispersed γ-Fe_2_O_3_ and CeO_2_, while the F_2g_ band characteristic of ceria was not detected. The Raman spectrum of 1Ce6Fe has broad bands with maxima at ~350 cm^−1^, ~500 cm^−1^, and ~670 cm^−1^. In the spectrum of maghemite (γ-Fe_2_O_3_) [44], three broad bands appear centered at ~350 cm^−1^, ~500 cm^−1^, and ~660 cm^−1^, corresponding to the vibrational modes E_g_, T_2g_, and A_1g_. Thus, according to the Raman spectroscopy data, the introduction of 25% Fe_2_O_3_ into the support (3Ce2Fe sample) significantly increases the defectiveness of CeO_2_, which is one of the reasons for its high activity in toluene oxidation.

The nature of the interaction of the supported Pt with the surface of the oxide supports plays a significant role in determining the catalytic activity. Figure 5 also shows the spectra of the supported Pt catalysts in comparison with the supports. For the Pt/CeO_2_ sample, the F_2g_ mode in the CeO_2_ structure does not change its position, which indicates the preservation of the cubic structure of fluorite CeO_2_. In the presence of platinum, an increase in the signal in the range of 500–650 cm^−1^ is observed, which is associated with the appearance of defects in the CeO_2_ structure and oxygen vacancies [35]. For the Pt/CeO_2_ sample, the appearance of vibrations corresponding to Pt-O-Ce and Pt-O bonds is also possible, overlapping with the vibration region of defective CeO_2_. Thus, Pt-O-Ce vibrations at 548–570 cm^−1^ are attributed to single-atom Pt^2+^ species on the CeO_2_ surface or to PtO_x_ clusters on the CeO_2_ surface [45]. The band at 656–660 cm^−1^ corresponds to Pt–O vibrations in the PtO_x_ clusters [46] or in PtO nanoparticles. According to Ref. [47], the presence of both bands indicates the formation of surface solid solutions of PtCeO_x_. This assumption is consistent with the TPR-H_2_ data (Figure 3). The value of I_D_/I_F2g_ for Pt/CeO_2_ increases to 0.0454 in comparison with pure CeO_2_ (0.0284).

In the Raman spectrum of Pt/3Ce2Fe, a shift in the main F_2g_ band to a shorter wavelength region of 452 cm^−1^ is observed, which indicates a decrease in the size of the CeO_2_ particles and is consistent with the XRD data [48]. The general increase in the background signal and the increase in the intensity ratio of the D/F_2g_ bands indicate an increase in the overall defectiveness of CeO_2,_ not only due to the introduction of iron oxide, but also Pt. A reflection corresponding to metallic platinum was detected in the XRD pattern of the Pt/3Ce2Fe sample. However, large Pt/PtO_x_ particles do not produce signals in the Raman spectra, but their presence can be indirectly judged by the disordering of the support lattice. In Ref. [49], a similar effect was observed for large Pt particles, which had a greater effect on the CeO_2_ defectiveness compared to small Pt clusters. The value of I_D_/I_F2g_ for Pt/3Ce2Fe is 0.4519, which is significantly higher than those for 3Ce2Fe (0.2364) and pure CeO_2_ (0.0284). Thus, both the addition of Fe and Pt significantly increases the defectiveness of CeO_2,_ with the respective growth of the content of oxygen vacancies.

For the Pt/1Ce6Fe catalyst, no additional bands appear in the Raman spectrum compared to the support; an increase in intensity is observed for the maxima at 352, 481, and 649 cm^−1^. For the Pt/Fe_2_O_3_ sample, the spectra of the iron oxide and Pt catalysts are virtually indistinguishable. Approaches to create oxygen vacancies in iron oxide were described in the limited literature; however, they require high-temperature treatments [50], the introduction of additional cations, such as Ca^2+^, Mg^2+^ [51], or ensuring strong metal/support interactions of Pt/Fe_2_O_3_ [52,53].

**Figure 6:**
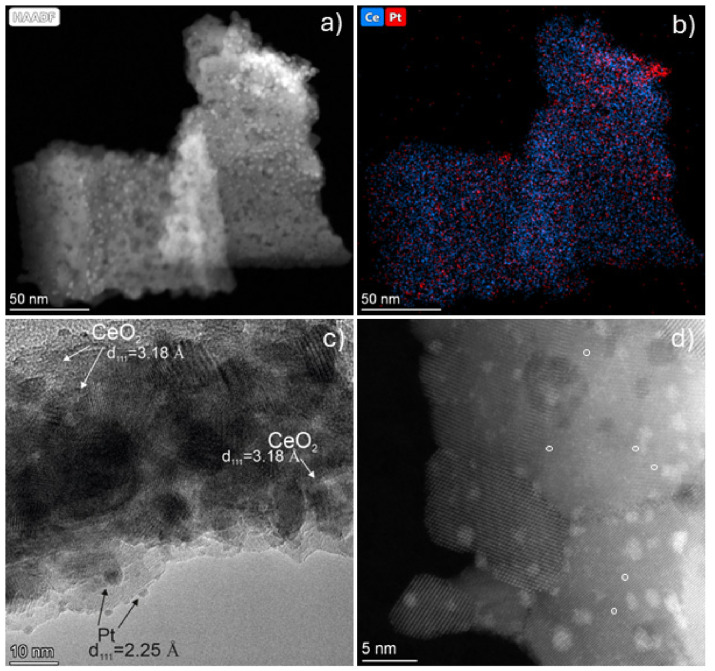
TEM images of Pt/CeO_2_: HAADF-STEM image of Pt/CeO_2_ catalyst (**a**) and corresponding EDX maps of Ce and Pt element distribution (**b**), HR TEM (**c**) and HAADF-STEM images (**d**) of Pt/CeO_2_ region, the circles indicate the single-atom Pt species.

The differences in the catalytic properties of the presented samples are also determined by the distribution and nature of the interaction between the supported Pt and the support surface. The TEM image (Figure 6a) shows that the structure of the Pt/CeO_2_ sample is represented by ceria agglomerates with Pt species distributed over the entire surface, according to EDX mapping data. The interplanar distances measured from the microdiffraction pattern correspond to the distances in CeO_2_ with a fluorite structure (Figure 6c). Numerous Pt particles of ~1.5–2 nm in size are observed on the surface of the polycrystalline particles. The particles have a flattened shape due to the interaction with the support. In addition, Figure 6d shows a high-magnification HR STEM image of a section of the Pt/CeO_2_ sample, in which platinum is present in the form of clusters and particles up to 1 nm in size, as well as single atoms (marked with circles). In addition, it is evident that platinum species are localized at the grain boundaries of CeO_2_.

**Figure 7:**
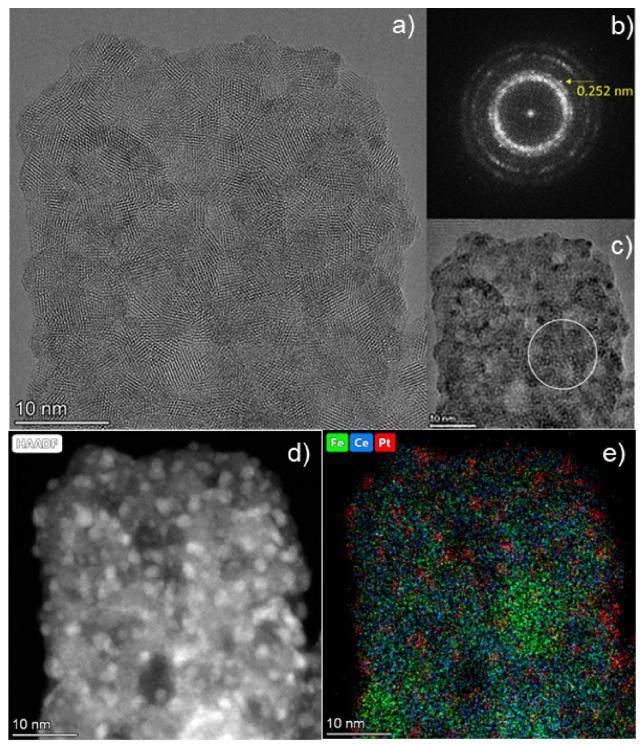
HRTEM image of Pt/3Ce2Fe (**a**), FFT pattern (**b**) and Fourier filtered image for the marked reflection (**c**), white circle shows the localization region of the iron oxide nanoparticle; HAADF-STEM image of Pt/3Ce2Fe (**d**) and corresponding EDX maps (**e**) of the elemental distributions (Fe, Ce, Pt).

Figure 7a shows the HRTEM image of the Pt/3Ce2Fe catalyst and the corresponding FFT pattern (Figure 7b), which, in addition to the rings associated with the CeO_2_ phase, contains a reflection that is absent in the CeO_2_ phase. The interplanar spacing corresponding to this reflection is 0.252 nm. Although the phase identification based on the interplanar spacing alone is difficult, such spacings exist in a number of iron oxide phases. For instance, for γ-Fe_2_O_3_ (311), d = 0.252 nm (No. 24-0081), and for Fe_3_O_4_ (311), d = 0.2532 nm (No. 19-0629). The image after the Fourier filtering for this reflection shows the localization region of the iron oxide nanoparticle (Figure 7c), which coincides with the area of increased Fe signal in the EDX mapping (Figure 7e). In the FFT, the reflections from the Pt nanoparticles are rather weak and practically invisible. This is due to both the small size of the particles and the disordered structure and dynamic behavior of the particles under the beam. Figure 7d visualizes individual Pt nanoparticles with a size of approximately 1.5–2 nm; however, many Pt particles are characterized by an amorphous structure due to their small size. According to the EDX map of element distribution (Figure 7e), Pt is distributed as dispersed clusters and particles over 3Ce2Fe support; however, it can be seen that the Pt concentration on the Fe-rich surface (green area) is lower, which may indicate the Pt distribution predominantly on the CeO_2_ surface. Also, this may be attributed to the Pt distribution over the FeO_x_ surface as single-atom species, but this cannot be confirmed by TEM in this mode because of the high mobility of dispersed Pt species under the electronic beam.

## 4. Discussion

**Figure 8:**
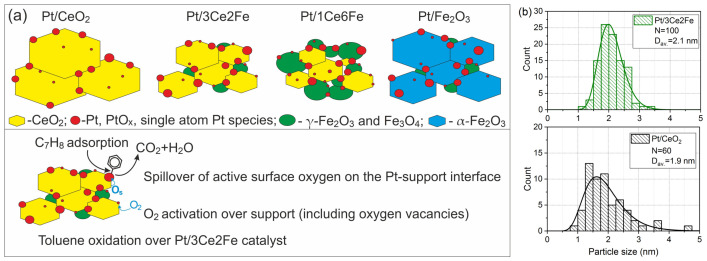
The schematic illustration of Pt/CeO_2_-Fe_2_O_3_ catalysts and a scheme of toluene oxidation over Pt/3Ce2Fe catalyst (**a**); Pt size distribution for Pt/CeO_2_ and Pt/3Ce2Fe catalysts (**b**) (calculated for the corresponding STEM images in Figure 6d and Figure 7d).

Thus, CeO_2_, Fe_2_O_3_, and the 3Ce2Fe and 1Ce6Fe mixed oxide systems were synthesized by citrate method using excess citric acid (molar ratio of metals to citric acid was 1:2). Mixed Ce-Fe oxides are characterized by the increased surface area in comparison with the individual CeO_2_ and Fe_2_O_3_ oxides that is linked with increased dispersion of CeO_2_ and FeO_x_ crystallites (Figure 8a). Both the Fe incorporation into the CeO_2_ structure and the formation of CeO_2_-FeO_x_ interfaces are observed. The Ce addition to Fe_2_O_3_ stabilizes highly dispersed γ-Fe_2_O_3_ and Fe_3_O_4_ phases in comparison with the predominant α-Fe_2_O_3_ phase for the pure Fe_2_O_3_ sample. The increased defectiveness of the CeO_2_ phase for 3Ce2Fe and 1Ce6Fe samples was shown by XRD and Raman. Pt deposition on the oxide support does not lead to significant changes in textural characteristics but significantly increases reducibility in the low-temperature region. Using the H_2_-TPR method, it is established that the Pt deposition on the surface reduces the surface reduction temperature of the oxide supports, which facilitates the involvement of active oxygen species in oxidation processes, including toluene oxidation, at relatively lower temperatures (50–250 °C). Platinum had the greatest effect on the surface reduction ability of the catalysts for the Pt/3Ce2Fe and Pt/1Ce6Fe mixed samples. Platinum on the surface of these catalysts is present in the form of small PtO_x_ particles, and the hydrogen consumption maximum corresponding to the reduction in Fe_2_O_3_ to Fe_3_O_4_ disappears after the Pt deposition. For 3Ce2Fe and Pt/3Ce2Fe samples. Raman spectroscopy shows a significant increase in the defectiveness of the CeO_2_ structure due to the introduction of Pt.

In the toluene oxidation reaction, Pt/CeO_2_ and Pt/3Ce2Fe are shown to exhibit comparable activity, with 50% conversion temperatures differing only slightly, i.e., 163 and 167 °C, respectively. Toluene conversion over Pt/Fe_2_O_3_ begins at a higher temperature compared to Pt/CeO_2_ and Pt/3Ce2Fe, while Pt/3Ce2Fe is characterized by the lowest activity. Despite the activity of Pt/CeO_2_ and Pt/3Ce2Fe being similar, the fact that there was more significant activity growth for Pt/3Ce2Fe in comparison with 3Ce2Fe is of interest, as the information about the structure–activity relationships may be revealed. Figure 8a shows the schematic toluene oxidation over Pt/3Ce2Fe catalysts, which includes adsorption of toluene and O_2_ on the catalyst surface, spillover of active oxygen through the surface to the Pt–support interface, and oxidation of the adsorbed toluene with this oxygen with the release of CO_2_ and water. What are possible reasons for the high activity of the Pt/3Ce2Fe catalyst?

(1) ***Increased surface area*** (42 m^2^/g) in comparison with Pt/CeO_2_ (19 m^2^/g) and Pt/Fe_2_O_3_ (31 m^2^/g) catalysts, which is favorable for toluene and O_2_ adsorption and stabilization of dispersed Pt species. However, the Pt/1Ce6Fe catalyst with the highest surface area (97 m^2^/g) is characterized by the lowest catalytic activity. Probably, the FeO_x_ phases are predominant on the 1Ce6Fe surface support, while the CeO_2_ surface is more active in both O_2_ activation and toluene adsorption [26]. Thus, there is no clear correlation between the surface area and catalytic properties, but probably the surface area of the CeO_2_ component in the mixed systems is crucial. Probably, the surface area of CeO_2_ in the 3Ce2Fe support is relatively high in comparison with the pure CeO_2_ because of the increased dispersion of the CeO_2_ phase.
(2) ***Platinum dispersion***. The high surface area of the 3Ce2Fe support may lead to the increased dispersion of the Pt species. To estimate this, the particle size distributions were calculated for Pt/CeO_2_ and Pt/3Ce2Fe catalysts (Figure 8b). Both catalysts are characterized by a relatively thin particle size distribution from 1 to 3 nm with an average particle size of 1.9 and 2.1 nm, respectively. The insignificant increase in the particle size is observed for the Pt/3Ce2Fe catalyst; however, the contribution of small clusters and single-atom species cannot be estimated. Thus, the clear correlation between the size of Pt particles and catalytic activity cannot be found for these catalysts.
(3) ***Increased defectiveness and increased amount of oxygen vacancies***, which are shown by XRD and Raman. The increased defectiveness leads to increased activity in O_2_ activation. However, the highest defectiveness is observed for the Pt/1Ce6Fe catalyst. Probably, the high dispersion of the CeO_2_ crystallites and their stabilization by amorphous FeO_x_ particles (scheme in Figure 8a) lead to a low oxygen mobility. Spillover of the surface oxygen plays an important role in oxidative processes, including low- and middle-temperature VOCs oxidation. Thus, a small amount of Fe dopant leads to Fe intercalation into the CeO_2_ structure, increasing the defectiveness and reaction ability of CeO_2_; however, the moderate amounts of Fe lead to the formation of the CeO_2_-FeO_x_ interface with the corresponding rise in the surface area and CeO_2_ dispersion. A high amount of Fe (1Ce6Fe) significantly blocks the CeO_2_ particles and decreases their reaction ability.
(4) ***Pt–support interface***. The toluene oxidation occurs on both surfaces of the support (CeFe oxide is also active, Figure 4b) and over the Pt species, and the activity of the Pt catalysts is significantly higher. Therefore, the Pt–support interface plays an important role in the cooperation of active species of Pt and support, as well as oxygen spillover from the support to adsorbed toluene. The number of this interface and the effectiveness of oxygen spillover through this interface can be estimated from the TPR-H_2_ results. The low-temperature reduction in the support surface is linked to both H_2_ activation over the Pt species with their spillover to the Pt–support interface, and the spillover of the support surface oxygen (O_s_) to this interface. For Pt/CeO_2_ catalysts (Figure 3), the simultaneous reduction in the PtO_x_ species and surface CeO_2_ is observed at temperatures of 50–250 °C, which indicates the formation of the Pt–CeO_2_ interface. However, the high intensity of the peaks at 250–550 °C is related to the reduction in the CeO_2_ surface without contact with Pt. Thus, the Pt–CeO_2_ interface is characterized by the high effectiveness of cooperation of Pt and CeO_2_ species, but the perimeter or area of this Pt–CeO_2_ interface boundary is relatively low. For the Pt/Fe_2_O_3_ catalyst, the shift in the peak related to the Fe_2_O_3_ reduction to Fe_3_O_4_ from 250 to 370 °C to 220–290 °C in comparison with the Fe_2_O_3_ support is observed. This indicates the presence of an interface between Pt and Fe_2_O_3_; however, a relatively low shift in this peak indicates the relatively low oxygen mobility of surface oxygen in Fe_2_O_3_. The highest low-temperature reducibility is observed for the Pt/3Ce2Fe catalyst: the amount of consumed H_2_ at 50–250 °C is rather high in comparison with other catalysts and is attributed to the reduction in the dispersed PtO_x_ species and surface species of CeO_2_ and FeO_x_. Thus, the cooperation of active Pt species with the active surface oxygen is more pronounced due to both the developed Pt–support interface and oxygen surface mobility.

Thus, it can be concluded that the high activity of the Pt/3Ce2Fe catalysts is linked to many factors, including Fe incorporation into the CeO_2_ structure and the formation of an optimal number of CeO_2_-FeO_x_ interfaces with the respective rise in the specific surface area, etc. The features of the Pt–support interface, including the length of this interface and the effectiveness of oxygen spillover through this interface, play a key role in the cooperation of active species of Pt and support and the rise in activity in the toluene oxidation.

The catalytic activity of the Pt/CeO_2_-Fe_2_O_3_ catalyst was compared with that of other Pt catalysts; the results are summarized in Table 2. The activity is comparable with that of other supported Pt catalysts. In our opinion, the catalysts with higher activity may be found during the optimization of the Ce/Fe ratio between Pt/CeO_2_ and Pt/3Ce2Fe catalysts.

## 5. Conclusions

In this study, the CeO_2_, 3Ce2Fe, 1Ce6Fe, and Fe_2_O_3_ oxides were prepared using the citrate method. For mixed Ce-Fe supports, an increase in the dispersion of the oxide phases and the specific surface area was demonstrated. The XRD analysis showed that with the ceria addition, the iron oxide crystallized into γ-Fe_2_O_3_, while the addition of Fe to CeO_2_ leads to intercalation of Fe into the CeO_2_ structure and formation of CeO_2_-FeO_x_ interfaces that lead to an increase in the defectiveness and reaction ability of CeO_2_ particles. Supported Pt/CeO_2_-Fe_2_O_3_ catalysts are active in the total oxidation of toluene. The formation of Pt-support interphase boundaries as well as high defectiveness and surface oxygen mobility of support were crucial for the low-temperature activity of the catalyst. The high activity growth of the Pt/3Ce2Fe catalysts in comparison with respective 3Ce2Fe oxide support in toluene oxidation resulted from a combination of factors, including the incorporation of Fe into the CeO_2_ structure, the formation of an optimal amount of CeO_2_–FeO_x_ interfaces accompanied by an increase in specific surface area, and the reaction ability of the Pt–support interface. Among these, the length of the Pt–support interface and the efficiency of oxygen spillover across it played a key role in facilitating synergy between Pt and the active species of support, thereby boosting the catalytic performance. The next optimization of catalyst composition between the Pt/CeO_2_ and Pt/3Ce2Fe catalysts is necessary to find an optimal ratio between Fe incorporation into CeO_2_ and formation of CeO_2_-FeO_x_ interfaces. In addition, the stability test and resistance of catalysts toward moisture and poisons (S, Cl, etc.) are necessary for optimal catalysts to estimate the possibility of their application for toluene oxidation in real conditions.

## Figures and Tables

**Table 1:** Textural characteristics and phase composition of Ce-Fe supports and Pt catalysts based on them.

Sample	Phase Composition	d (CSR) (nm)	a (Å)	Δd/d × 10^3^	S_BET_ (m^2^/g)	V_pore_ (cm^3^/g)
CeO_2_	CeO_2_	16	5.4047	1.147	19	0.049
2 Pt/CeO_2_	CeO_2_	17	5.4032	0.389	19	0.049
3Ce2Fe	CeO_2_	9	5.4134	6.867	48	0.056
2 Pt/3Ce2Fe	CeO_2_	9	5.4147	6.532	42	0.048
1Ce6Fe	CeO_2_	9	5.4184	3.907	96	0.252
	γ-Fe_2_O_3_	16	a = 8.3662 c = 25.1340	0.649		
2 Pt/1Ce6Fe	CeO_2_	9	5.3933	5.929	97	0.240
	γ-Fe_2_O_3_	16	a = 8.3446 c = 25.1340	2.306		
Fe_2_O_3_	α-Fe_2_O_3_	36	a = 5.0302 c = 13.7420	1.067	32	0.261
	γ-Fe_2_O_3_	17	a = 8.3898 c = 24.9778	2.424		
2 Pt/Fe_2_O_3_	α-Fe_2_O_3_	44	a = 5.0336 c = 13.7498	0.853	31	0.220
	γ-Fe_2_O_3_	24	a = 8.3258 c = 25.1340	1.357		

**Table 2:** Catalytic properties of Pt catalysts in toluene oxidation.

Catalysts	Pt Content, %	Reaction Composition	WHSV or GHSV	T_50_ (°C)	Ref.
Pt/CeO_2_-HA	0.71	100 ppm toluene,	60,000 mL·g_cat_^−1^·h^−1^	138	[16]
Pt/CeO_2_	0.41	1000 ppm toluene, air in balance	15,000 mL·g^−1^·h^−1^	131	[54]
Pt/CeO_2_-0.5	1	1000 ppm toluene, air in balance	54,000 mL·g_cat_^−1^·h^−1^	155	[49]
Pt/CeO_2_	2	1000 ppm toluene, 20% O_2_, N_2_ in balance	30,000 h^−1^	163	This work
Pt-CeO_2_	not specified	1000 ppm toluene, 20% O_2_, N_2_ in balance	60,000 mL·g_cat_^−1^·h^−1^	151	[55]
Pt-Fe_3_Ce_1_	2	50 ppm toluene, air in balance,	36,000 mL·g_cat_^−1^·h^−1^	188	[24]
Pt/3Ce2Fe	2	1000 ppm toluene, 20% O_2_, N_2_ in balance	30,000 h^−1^	167	This work
Pt/1Ce6Fe	2	1000 ppm toluene, 20% O_2_, N_2_ in balance	30,000 h^−1^	214	This work
Pt/CuO-Fe_3_O_4_	1	100 ppm, air in balance	60,000 mL·g^−1^·h^−1^	168	[56]
Pt/Fe_2_O_3_	2	1000 ppm toluene, 20% O_2_, N_2_ in balance	30,000 h^−1^	195	This work
Pt/Fe_3_O_4_	1	100 ppm, air in balance	60,000 mL·g^−1^·h^−1^	148	[56]
Pt_1_Fe_0.79_/Mn_2_O_3_	0.62 Pt (0.14 Fe)	1000 ppm toluene, 20% O_2_, N_2_ in balance	40,000 mL·g_cat_^−1^·h^−1^	176	[57]
Pt/Al_2_O_3_	0.2	2000 ppm toluene, air in balance	3000 h^–1^	147	[58]
7CuO/CeO_2_	-	900 ppm toluene and 21% O_2_, Ar in balance	23,000 h^−1^	240	[59]
Pd_2_Pt_1_/SiO_2_	Total 0.5	1000 ppm toluene, air in balance	24,000 h^−1^	160	[60]
2%Pt/MS	2	100 ppm	not specified	137	[61]

## Data Availability

Data available on request.

## References

[1] Zhou X., Zhou X., Wang C., Zhou H. (2023). Environmental and human health impacts of volatile organic compounds: A perspective review. Chemosphere.

[2] Zhang Z., Jiang Z., Shangguan W. (2016). Low-temperature catalysis for VOCs removal in technology and application: A state-of-the-art review. Catal. Today.

[3] Shan C., Wang Y., Li J., Zhao Q., Han R., Liu C., Liu Q. (2023). Recent Advances of VOCs Catalytic Oxidation over Spinel Oxides: Catalyst Design and Reaction Mechanism. Environ. Sci. Technol..

[4] Yi J., Liu J., Gao B., Bo L., Cao L., Sillanpää M. (2025). The comprehensive review of catalysts for catalytic oxidation of volatile organic compounds. J. Environ. Chem. Eng..

[5] Zang M., Zhao C., Wang Y., Chen S. (2019). A review of recent advances in catalytic combustion of VOCs on perovskite-type catalysts. J. Saudi Chem. Soc..

[6] Ren Y., Dong C., Song C., Qu Z. (2024). Spinel-Based Catalysts That Enable Catalytic Oxidation of Volatile Organic Compounds. Environ. Sci. Technol..

[7] Luo X., Xue Y., Xu L., Lian L., Wu C., Cui Y., Zou W., Dong L., Gao F., Chen M. (2025). Comparative Study of Catalytic Oxidation of Toluene over Porous Metal Oxide Catalysts Derived from (Mn, Ce, Co)-MOFs. Inorg. Chem..

[8] Xin Y., Zhang H., Liu J., Wang J., An X., Wu X. (2024). Achieving deep oxidation of toluene over CoMnO_x_ catalyst: Insight into the collaboration of Co_3_O_4_ and MnO_x_ via layered double hydroxides (LDHs) precursor template. J. Environ. Chem. Eng..

[9] Li Y., Chen T., Zhao S., Wu P., Chong Y., Li A., Zhao Y., Chen G., Jin X., Qiu Y. (2022). Engineering Cobalt Oxide with Coexisting Cobalt Defects and Oxygen Vacancies for Enhanced Catalytic Oxidation of Toluene. ACS Catal..

[10] Lu Y., Deng H., Pan T., Wang L., Zhang C., He H. (2022). Interaction between noble metals (Pt, Pd, Rh, Ir, Ag) and defect-enriched TiO2 and its application in toluene and propene catalytic oxidation. Appl. Surf. Sci..

[11] Salaev M.A., Salaeva A.A., Kharlamova T.S., Mamontov G.V. (2021). Pt–CeO_2_-based composites in environmental catalysis: A review. Appl. Catal. B Environ..

[12] Xu M., Peng M., Tang H., Zhou W., Qiao B., Ma D. (2024). Renaissance of Strong Metal–Support Interactions. J. Am. Chem. Soc..

[13] Ren Q., Zhao X., Zhou L., Song L., Tian J., Nie J., Liu P., Ye D., Wang Z. (2025). Regulating metal–support interaction of Pt/CeO_2_ catalysts via alkali metal modification for efficient toluene oxidation. J. Mater. Chem. A.

[14] Salaev M.A., Xiong H., Cortés Corberán V., Liotta L.F., Vodyankina O.V. (2025). Synergistic effects in heterogeneous catalysis: Status and perspectives. Mater. Today Chem..

[15] Grabchenko M.V., Mikheeva N.N., Mamontov G.V., Salaev M.A., Liotta L.F., Vodyankina O.V. (2018). Ag/CeO_2_ Composites for Catalytic Abatement of CO, Soot and VOCs. Catalysts.

[16] Yan D., Li X., Zhong J., Ren Q., Zeng Y., Gao S., Liu P., Fu M., Ye D. (2024). Tuning the Metal–Support Interaction by Modulating CeO_2_ Oxygen Vacancies to Enhance the Toluene Oxidation Activity of Pt/CeO_2_ Catalysts. Inorg. Chem..

[17] Zhang L., Xue X., Guo T., Bi L., Hu T., Tan L., Zhang X., Jiang J., Hong K., Zhang Q. (2021). Creation of oxygen vacancies to activate Fe_2_O_3_ photoanode by simple solvothermal method for highly efficient photoelectrochemical water oxidation. Int. J. Hydrogen Energy.

[18] Gao R., Wang J., Huang Z.F., Zhang R., Wang W., Pan L., Zhang J., Zhu W., Zhang X., Shi C. (2021). Pt/Fe_2_O_3_ with Pt–Fe pair sites as a catalyst for oxygen reduction with ultralow Pt loading. Nat. Energy.

[19] Han C., Yoko A., Taufik A., Ohara S., Nishibori M., Ninomiya K., Kiuchi H., Harada Y., Adschiri T. (2025). High Oxygen Storage Capacity of Ultrasmall Mn-Doped CeO_2_ Nanoparticles via Enhanced Local Distortion and Mn(II) Lattice Substitution. Chem. Mater..

[20] Fan J., Niu X., Teng W., Zhang P., Zhang W., Zhao D. (2020). Highly dispersed Fe–Ce mixed oxide catalysts confined in mesochannels toward low temperature oxidation of formaldehyde. J. Mater. Chem. A.

[21] Dou B., Yang D., Kang T., Xu Y., Hao Q., Bin F., Xu X. (2021). Morphology effects of CeO_2_-ZrO_2_ on the catalytic performance of CuO/CeO_2_-ZrO_2_ for toluene oxidation. Carbon Resour. Convers..

[22] Tian Y., Yang H., Wang F., Ning P., Li K., Li K. (2026). Construction of α- and γ- mixed phase Fe_2_O_3_ enhances the release of lattice oxygen thus achieving selective catalytic oxidation of H_2_S at low temperature. J. Catal..

[23] Guan Y., Zhang G., Wang R., Wang Y., Liu Y. (2024). Study on the synergistic effect and oxygen vacancy of CeO_2_/Fe_2_O_3_ oxygen carrier for improving reactivity in carbon monoxide chemical looping combustion. Fuel.

[24] Zhou Y., He J., Chen D., Li X., Wang Y., Xiao J., Li N., Xu Q., Li H., He J. (2021). Flower-like Pt/Fe_2_O_3_–CeO_2_ Catalysts for Highly Efficient Low-Temperature Catalytic Oxidation of Toluene. Ind. Eng. Chem. Res..

[25] Vasilchenko D., Topchiyan P., Berdyugin S., Filatov E., Tkachev S., Baidina I., Komarov V., Slavinskaya E., Stadnichenko A., Gerasimov E. (2019). Tetraalkylammonium Salts of Platinum Nitrato Complexes: Isolation, Structure, and Relevance to the Preparation of PtO_x_/CeO_2_ Catalysts for Low-Temperature CO Oxidation. Inorg. Chem..

[26] Chernykh M.V., Mikheeva N.N., Mamontov G.V. (2024). Unexpected strong toluene chemisorption over Ag/CeO_2_ catalysts for total toluene oxidation. Colloids Surf. A Physicochem. Eng. Asp..

[27] Bao H., Qian K., Fang J., Huang W. (2017). Fe-doped CeO_2_ solid solutions: Substituting-site doping versus interstitial-site doping, bulk doping versus surface doping. Appl. Surf. Sci..

[28] Cheng Z., Fu Q., Duan H., Cui Z., Xue Y., Zhang W. (2019). Size-Dependent Thermodynamics of Structural Transition and Magnetic Properties of Nano-Fe_2_O_3_. Ind. Eng. Chem. Res..

[29] Liu R., Li S., Du J., Yang Y., Xu W., Zhu T. (2022). Effect of CeO_2_ and Pt introduction on the structure and performance of Fe_2_O_3_ for Hg^0^ removal. Fuel.

[30] Abarzúa G., Roa S., Espinoza N., Cobo R., Sanhueza F. (2025). Synthesis of Fe_2_O_3_-CeO_2_ composites by fast combustion method: A study on citric acid, precursors concentration ratio and annealing temperature effects. Chem. Phys. Lett..

[31] Hojo H., Hirota K., Ito S., Einaga H. (2023). Reduction Mechanism for CeO_2_ Revealed by Direct Observation of the Oxygen Vacancy Distribution in Shape-Controlled CeO_2_. Adv. Mater. Interfaces.

[32] Zieli’nski J., Zglinicka I., Znak L., Kaszkur Z. (2010). Reduction of Fe_2_O_3_ with hydrogen. Appl. Catal. A.

[33] Taratayko A.V., Kuznetsov T.A., Kozhina M.V., Mamontov G.V. (2025). Influence of a Silver Precursor Introducing Method on the Properties of Magnetically Recoverable Ag/FeO_x_ Catalysts in 4-Nitrophenol Reduction. Russ. J. Inorg. Chem..

[34] Zhang L., Spezzati G., Muravev V., Verheijen M.A., Zijlstra B., Filot I.A.W., Su Y.Q., Chang M.W., Hensen E.J.M. (2021). Improved Pd/CeO_2_ Catalysts for Low-Temperature NO Reduction: Activation of CeO_2_ Lattice Oxygen by Fe Doping. ACS Catal..

[35] Lee J., Ryou Y., Chan X., Kim T.J., Kim D.H. (2016). How Pt Interacts with CeO_2_ under the Reducing and Oxidizing Environments at Elevated Temperature: The Origin of Improved Thermal Stability of Pt/CeO_2_ Compared to CeO_2_. J. Phys. Chem. C.

[36] Lashina E.A., Slavinskaya E.M., Stonkus O.A., Boronin A.I. (2024). Abnormally narrow peaks in TPR-H_2_ over Pt/CeO_2_: Experiment and mathematical modelling. Int. J. Hydrogen Energy.

[37] Bugrova T.A., Kharlamova T.S., Svetlichnyi V.A., Savel’eva A.S., Salaev M.A., Mamontov G.V. (2021). Insights into formation of Pt species in Pt/CeO_2_ catalysts: Effect of treatment conditions and metal-support interaction. Catal. Today.

[38] Marić I., Dražić G., Radin E., Peter R., Škrabić M., Jurkin T., Pustak A., Baran N., Mikac L., Ivanda M. (2023). Impact of platinum loading and dispersion on the catalytic activity of Pt/SnO_2_ and Pt/α-Fe_2_O_3_. Appl. Surf. Sci..

[39] Ma L., Chen X., Li J., Chang H., Schwank J.W. (2020). Electronic metal-support interactions in Pt/FeO_x_ nanospheres for CO oxidation. Catal. Today.

[40] Pathak V., Lad P., Thakkar A.B., Thakor P., Deshpande M.P., Pandya S. (2023). Synthesis, characterization and applications of cubic fluorite cerium oxide nanoparticles: A comprehensive study. Result. Surf. Interfaces.

[41] Wang H., Tsilomelekis G. (2020). Catalytic performance and stability of Fe-doped CeO_2_ in propane oxidative dehydrogenation using carbon dioxide as an oxidant. Catal. Sci. Technol..

[42] Dorofeeva N.V., Savel’eva A.S., Grabchenko M.V., Chernykh M.V., Bugrova T.A., Mamontov G.V., Salaev M.A. (2024). Ce−Zr−Mn Oxide Catalysts for Soot Combustion: The Role of Preparation Method. J. Phys. Chem. C.

[43] Shinde T.B., Mharsale N.N., Raut P.S., Patil G.E., Kapadnis K.H. (2025). Heterojunction p-n type α-Fe_2_O_3_/CeO_2_ composite for photocatalytic dye degradation. J. Indian Chem. Soc..

[44] Testa-Anta M., Ramos-Docampo M.A., Comesaña-Hermo M., Rivas-Murias B., Salgueiriño V. (2019). Raman spectroscopy to unravel the magnetic properties of iron oxide nanocrystals for bio-related applications. Nanoscale Adv..

[45] Stadnichenko A.I., Slavinskaya E.M., Stonkus O.A., Boronin A.I. (2024). Low-Temperature CO Oxidation by the Pt/CeO_2_ Based Catalysts. ChemCatChem.

[46] Kim Y., Oh D.G., Cho S.J., Khivantsev K., Kwak J.H. (2024). Catalytic behavior of Pt single-atoms supported on CeO_2_. Catal. Today.

[47] Slavinskaya E.M., Stadnichenko A.I., Muravyov V.V., Kardash T.Y., Derevyannikova E.A., Zaikovskii V.I., Stonkus O.A., Lapin I.N., Svetlichnyi V.A., Boronin A.I. (2018). Transformation of a Pt–CeO_2_ Mechanical Mixture of Pulsed-Laser-Ablated Nanoparticles to a Highly Active Catalyst for Carbon Monoxide Oxidation. ChemCatChem.

[48] Boronin A.I., Slavinskaya E.M., Figueroba A., Stadnichenko A.I., Kardash T.Y., Stonkus O.A., Fedorova E.A., Muravev V.V., Svetlichnyi V.A., Bruix A. (2021). CO oxidation activity of Pt/CeO_2_ catalysts below 0 °C: Platinum loading effects. Appl. Catal. B-Environ..

[49] Peng R., Li S., Sun X., Ren Q., Chen L., Fu M., Wu J., Ye D. (2018). Size effect of Pt nanoparticles on the catalytic oxidation of toluene over Pt/CeO_2_ catalysts. Appl. Catal. B-Environ..

[50] Wang J., Leng X., Kan S., Cui Y., Bai J., Xu L. (2024). Oxygen vacancies boosted photoelectrochemical performance of α-Fe_2_O_3_ photoanode via butane flame annealing. J. Alloys Compd..

[51] Wang S., Meng C., Bai Y., Wang Y., Liu P., Pan L., Zhang L., Yin Z., Tang N. (2022). Synergy Promotion of Elemental Doping and Oxygen Vacancies in Fe_2_O_3_ Nanorods for Photoelectrochemical Water Splitting. ACS Appl. Nano Mater..

[52] Zhang S., Chang X., Zhou L., Liu X., Zhang J. (2024). Stabilizing Single-Atom Pt on Fe_2_O_3_ Nanosheets by Constructing Oxygen Vacancies for Ultrafast H_2_ Sensing. ACS Sens..

[53] Li Z., Dai S., Ma L., Qu Z., Yan N., Li J. (2021). Synergistic interaction and mechanistic evaluation of NO oxidation catalysis on Pt/Fe_2_O_3_ cubes. Chem. Eng. J..

[54] Zhang Y., Wu C., Wang Z.h., Ji J., Wan H., Zou W., Tong Q., Sun J., Dong L., Chen Y.W. (2022). Enhanced low-temperature catalytic performance for toluene combustion of CeO2-supported Pt-Ir alloy catalysts. Appl. Surf. Sci..

[55] Xiao M., Han D., Yang X., Tchindad N.T., Dud L., Guo Y., Wei Y., Yu X., Ge M. (2023). Ni-doping-induced oxygen vacancy in Pt-CeO2 catalyst for toluene oxidation: Enhanced catalytic activity, water-resistance, and SO_2_-tolerance. Appl. Catal. B-Environ..

[56] Chen M., Fu L., Zhu D., Huang Y., Li R., He S., Liu S., Lee S., Cao J. (2025). Promoting Low-Temperature Toluene Oxidation via Pt–O–Fe Interfacial Sites in a Pt/CuO–Fe_3_O_4_ Catalyst. Environ. Sci. Technol..

[57] Feng Y., Wei L., Liu Y., Dai H., Zhao Z., Deng J. (2023). Rapid supplement of active oxygen by constructing Pt-Fe alloy structure to improve catalytic stability for furniture paints industry VOCs removal. Sep. Purif. Technol..

[58] Zhou B., Ke Q., Wen M., Ying T., Cui G., Zhou Y., Gu Z., Lu H. (2023). Catalytic combustion of toluene on Pt/Al_2_O_3_ and Pd/Al_2_O_3_ catalysts with CeO_2_, CeO_2_–Y_2_O_3_ and La_2_O_3_ as coatings. J. Rare Earths.

[59] Yi X., Wu L., Dou B., Liang W., Kang R., Bin F., Li Y. (2026). High-efficiency toluene degradation by non-thermal plasma modified catalysts: Oxygen vacancy-induced reactive oxygen species cycling mechanism. Appl. Catal. B.

[60] Cui J., Cui Y., Tan J., Zhang H., Gu M., Huang L. (2024). Efficient catalytic oxidation of VOCs by a Pd-Pt/SiO_2_ catalyst: The cooperative catalysis of dual metal sites. J. Environ. Chem. Eng..

[61] Zhou M., Li S., Cao M., Wang T., Nie L., Li W., Zhao F., Chen Y. (2023). Enhanced hydrophobic microporous Pt/silica with high adsorption and catalytic oxidation for trace toluene removal. J. Environ. Chem. Eng..

